# A task-experienced partner does not help dogs be as successful as wolves in a cooperative string-pulling task

**DOI:** 10.1038/s41598-018-33771-7

**Published:** 2018-10-30

**Authors:** Sarah Marshall-Pescini, Camille Basin, Friederike Range

**Affiliations:** 1 Wolf Science Center, Messerli Research Institute, University of Veterinary Medicine, Vienna, Medical University of Vienna, University of Vienna, Veterinaerplatz 1, 1210 Vienna, Austria; 2 Comparative Cognition, Messerli Research Institute, University of Veterinary Medicine, Vienna, Medical University of Vienna, University of Vienna, Veterinaerplatz 1, 1210 Vienna, Austria

## Abstract

Although theories of domestication have suggested that dogs evolved a greater capacity for tolerant and cooperative behaviour compared to their wild wolf cousins, the differences between wolves’ and free-ranging dogs’ social ecology, with wolves relying more on conspecific cooperation than dogs, would rather predict the opposite. In a cooperative task involving joint action on a rope to pull a tray forward, wolves systematically outperformed dogs. The dogs’ failure appeared largely due to tolerance issues, i.e. one partner avoiding interacting with the apparatus, when the other was engaged with it, rather than cognitive limitations. To verify this, in the current study we trained the dominant partner to become an ‘expert’ on the task thereby potentially enhancing their understanding that they ‘needed the partner to succeed’. Indeed both the duration of co-action on the apparatus and the success rate of dyads composed of an expert and an inexperienced dog was higher than dyads composed of two inexperienced partners. Nevertheless the dogs’ performance was substantially poorer than that of wolf dyads with equivalent experience, highlighting that despite the facilitating effect of the ‘expert’, cooperation on this task did not come easily to dogs. For both dogs and wolves, cooperation was facilitated by the closeness of the affiliative bond between individuals, but opposite rank effects emerged. Dogs further apart in rank were more successful co-operators, whereas in wolves, animals closer in rank had a higher cooperative success. The results further highlight the importance of the different socio-ecologies of wolves and dogs in understanding their behaviour.

## Introduction

Cooperative problem solving, defined as two individuals coordinating their actions to successfully solve a task which cannot be solved alone, has been experimentally investigated in a wide variety of species and one of the most popular methods has been the ‘loose string task’, in which two individuals are given the possibility of simultaneously pulling on two rope ends to move a baited tray within reach (first used with chimpanzees^1–4^ macaques^5^; ravens^6^; rooks^7^, elephants^8^; grey parrots^9^; kea^10^). More complex variants of the task have been devised over time to further investigate the animals’ understanding of the ‘cooperative’ nature of the task. For example in a “delay condition”, the subject is released before the partner with the rationale being that, if the former understands that it needs the partner to succeed, they should wait to pull the rope until its arrival at the apparatus. Similarly, a ‘two-apparatus’ condition (first used with hyenas in a variation of the string-pulling task^11^), requires partners to coordinate in both ‘time and space’, since to succeed in obtaining food from both platforms, they need to move together to first one and then to the other apparatus. In general, whereas almost all species tested succeeded in solving the single apparatus, fewer were also able to negotiate setups in which waiting was involved (chimpanzees^3^, elephants^8^, kea^10^ and ravens^12^ succeeded but rooks^7^ and grey parrots^9^ did not).

Not surprisingly, social relationships have been repeatedly shown to play a crucial role in such cooperative interactions. Indeed, the closeness of social bonds (in terms of affiliative interactions exchanged) as well as tolerance around a food source and rank have been shown to affect cooperative success in string-pulling tasks in ravens^6^, chimpanzees^3^, hyenas^11^, macaques^5^ and wolves^13^. Furthermore, in the one study that investigated the issue, it was found that partner experience significantly affected success, with hyena dyads composed of a task-naïve and a task-savvy partner being more successful than dyads composed of two naïve individuals^11^.

Dogs are a particularly interesting species in which to investigate conspecific cooperation, since it has been suggested that during domestication, we selected for specific temperament traits such as tolerance towards both humans and conspecifics, thereby promoting dogs’ inclination for cooperative interactions^14–16^. In a cooperative string pulling task, after an initial individual training phase, pet dogs were able to coordinate their actions with a conspecific and a human partner in the test condition, and subjects were even capable of tolerating short delays (of approximately 2 seconds with conspecifics and in 20% of trials they could wait 15 seconds for the human partner) suggesting some understanding that they needed the partner to succeed^17^.

**Figure 1:**
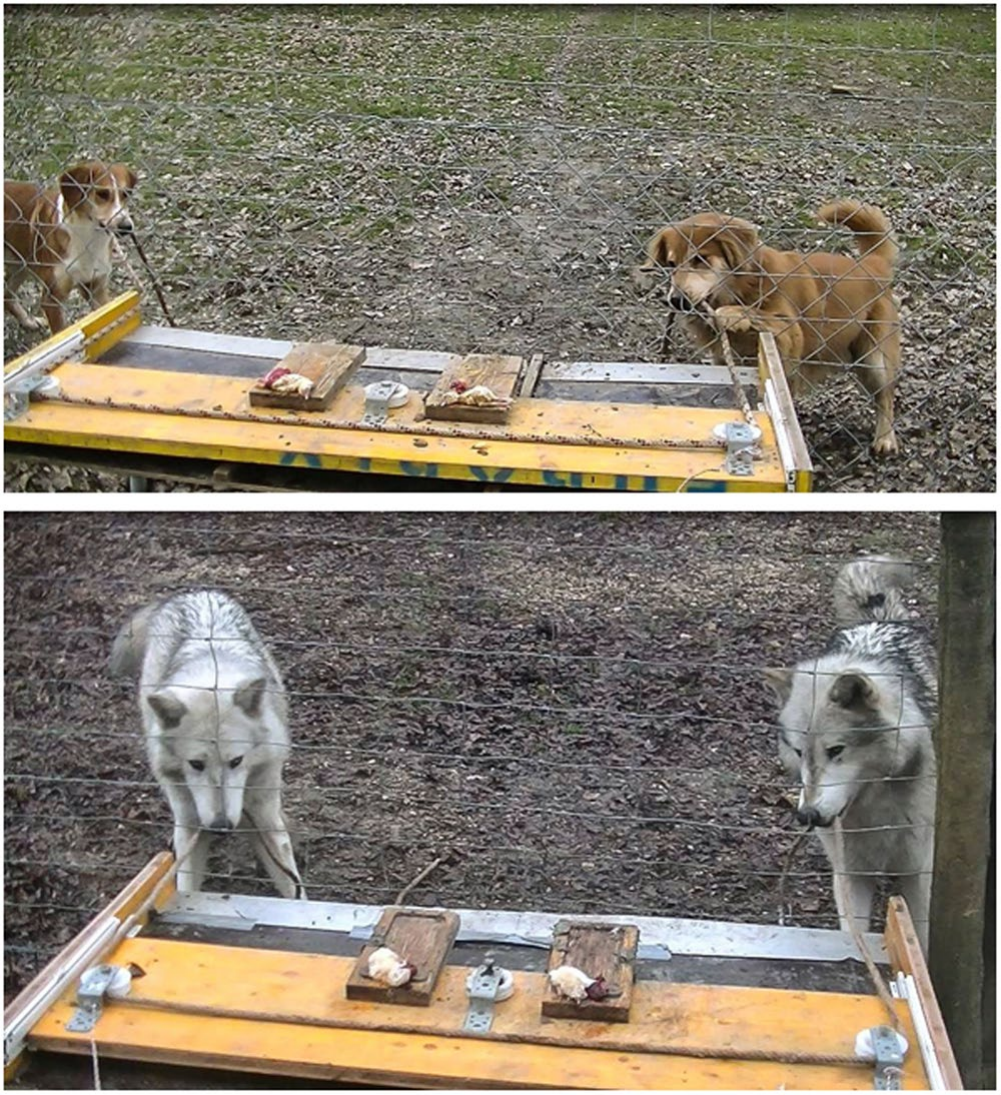
(**a,b**) Dogs & wolves working on the loose-string paradigm (photo credits: Camille Basin, Wolf Science Center).

The loose-string paradigm was also used to compare dogs with similarly raised wolves at the Wolf Science Center (WSC)^13^ (Fig. 1a,b). Differently from the previous loose-string study with pet dogs (and most other species except ravens), we first tested animals in a Spontaneous condition, in which both partners were presented with the task with no prior experience of the apparatus. Whereas five of the seven wolf dyads succeeded in at least one trial with success rates between 3% and 56% of trials, only one of the eight dog dyads succeeded and only in one trial. Following a training regime comparable to that by Ostojic and Clayton^17^, three of the four wolf dyads tested succeeded in 14–92% of trials, while only one of the six dog dyads succeeded in a single trial (3%). The dogs’ failure appeared to be linked to ‘tolerance’ issues rather than a lack of cognitive skills since dogs were less likely than wolves to interact with the apparatus at the same time. Usually one dog at a time interacted with the ropes and the tray, whilst the other typically stood by, or wandered off.

This avoidance of being active on the apparatus at the same time ultimately resulted in them almost never pulling the two rope ends together. Interestingly, this ‘conflict-avoidance strategy’ is in line with two other lines of research. In food-sharing studies, results show that the dominant dog will monopolize the food source and the subordinate individuals will maintain their distance without even trying to obtain the food, whereas in wolves subordinates will also feed^18,19^. Furthermore, in reconciliation studies it emerges that, whereas after a conflict wolf opponents will approach each other in a friendly manner to re-establish contact, dogs will rather maintain a greater distance from one another^20^. Similarly, results in the string-pulling task suggest that, because one partner avoids the apparatus on which the food is placed when the other is interacting with it, the likelihood of discovering that ‘cooperation’ is required is reduced.

**Figure 2:**
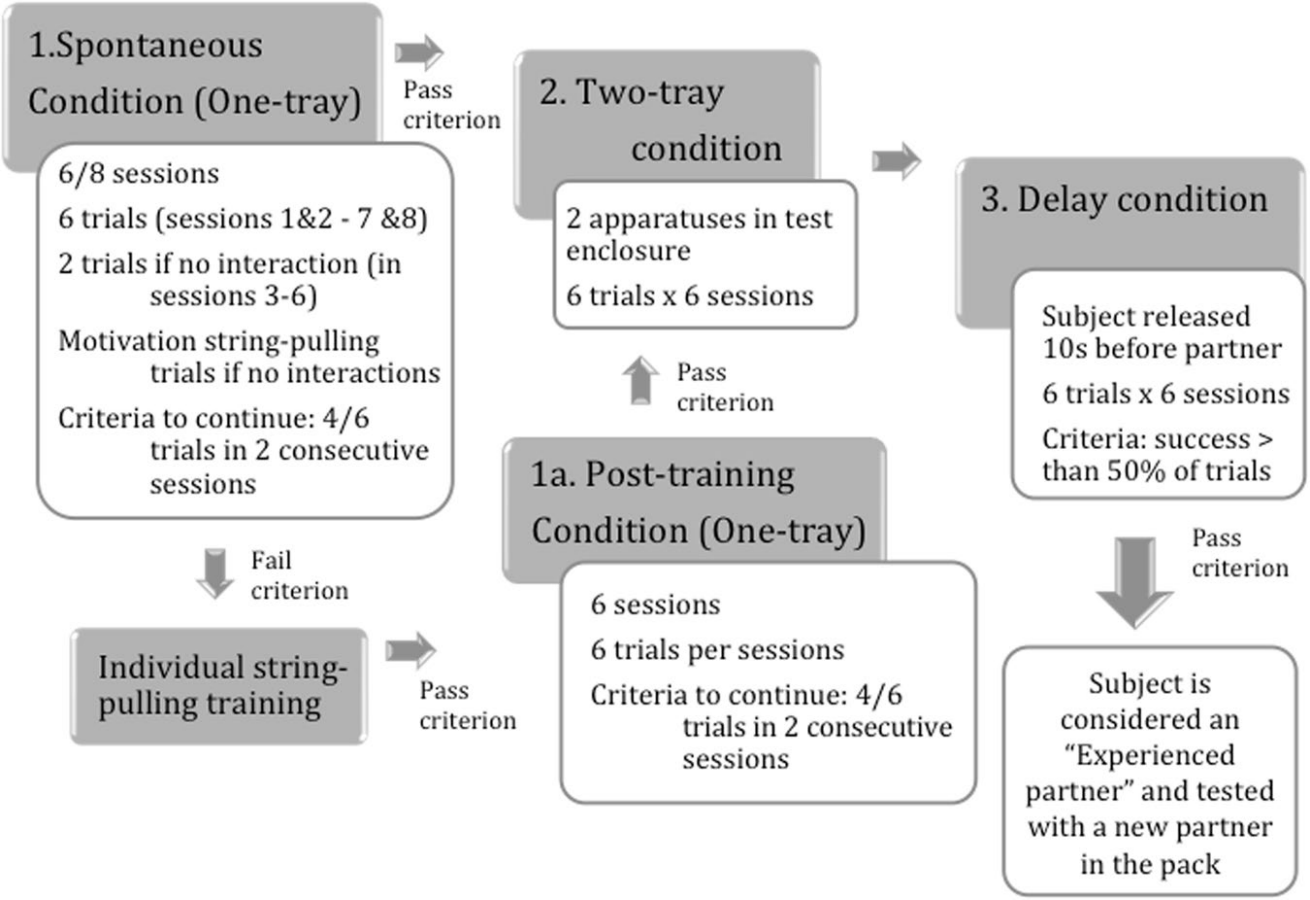
Schematic depiction of procedure (modified from Marshall-Pescini *et al*. 2017). Wolves were considered experienced once they had successfully progressed through the Two-tray and Delay condition. Dogs were tested only in the Spontaneous and Post-training condition, since they never passed the criterion to progress to the Two-tray condition.

In contrast to dogs, in wolf dyads, individuals did not hesitate to interact with the apparatus even when their partner was manipulating it and this facilitated the ‘discovery’ of the correct simultaneous rope-pulling response. Once cooperation was initiated, wolf dyads continued to be highly successful also when two apparatuses were presented (Two-tray condition) and when the subject was released 10 seconds prior to the partner (Delay condition) (Fig. 2). Because of dogs’ failure in the one-tray version of the task, we were unable to test them in the more complex two-tray and delay conditions.

The wolf and dog dyads compared in Marshall-Pescini *et al*.’s study^13^ were composed of members with little to no experience of the task i.e. either both task naïve, or with some prior unsuccessful experience with another partner. However, in hyenas, dyads’ success rates increased when they were composed of an experienced dominant individual and a naïve submissive partner^11^, potentially because an ‘experienced’ dominant partner that understands the need for the other, may be more tolerant towards the subordinate in a context involving a limited food resource.

Therefore in the current study (based on the same dataset of^13^), we compared wolf dyads that were composed of two partners that had had no prior success in the task (inexperienced dyads) with ‘mixed’ experienced dyads composed of one inexperienced and one experienced dominant partner i.e. a wolf that had successfully solved the task and had waited in the delay condition for its ‘previous’ partner, therefore presumably had some understanding of the need for the partner.

**Figure 3:**
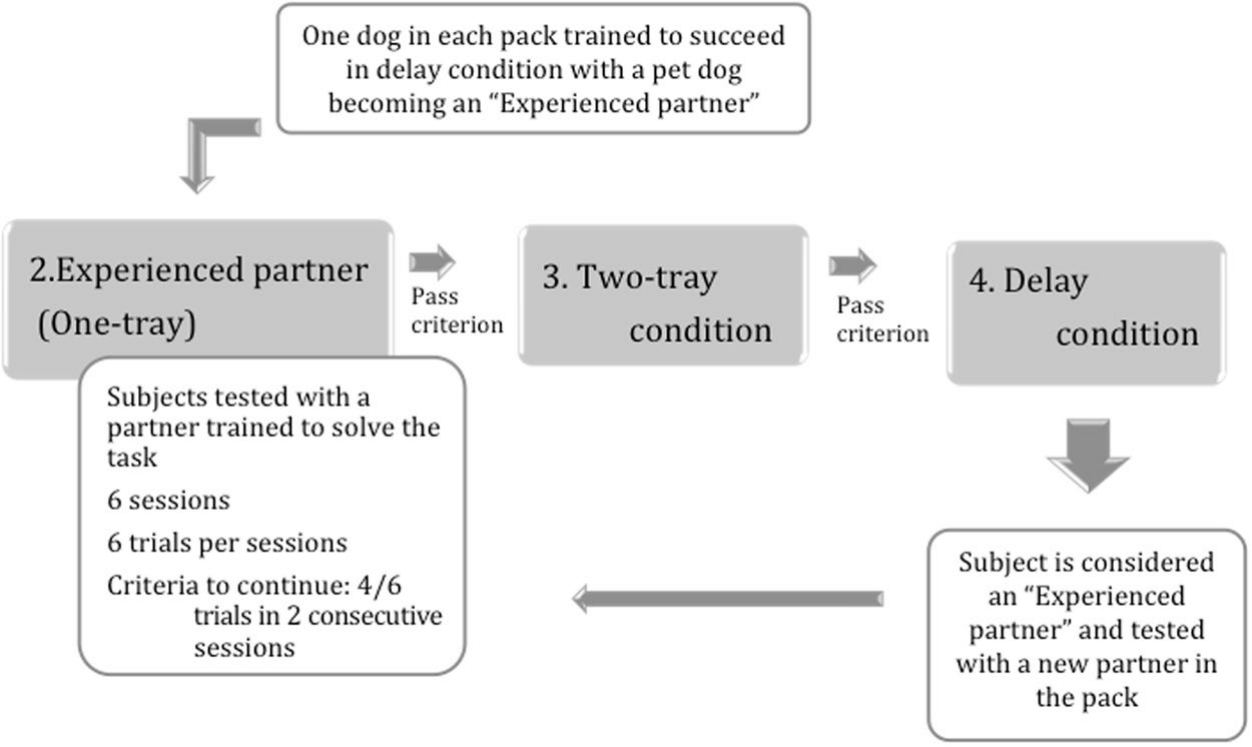
Schematic depiction of procedure for dogs in the current study in which they were tested with an experienced partner (i.e. with a dog trained to solve the task). If they met criterion, they could then progress to the Two-tray condition and Delay condition.

More importantly, we engineered this condition for dogs as well. In each pack, we trained the highest-ranking dog with a stooge pet dog to succeed and also learn to wait for the partner (for 30 seconds) thus becoming an ‘expert’. We then paired the dominant ‘expert’ dog with a pack mate consisting either of a dog that had failed in the previous conditions of the task or a dog with no prior experience of the task (‘mixed’ dyads- Fig. 3). As well as evaluating their success rate, we also looked at the details of their behaviour on the apparatus in the first session. If dominant expert partners are more ‘tolerant’ (because they better understand the role of the other), then in ‘mixed’ dyads we expect to see significantly more simultaneous interaction by both animals on the apparatus than in ‘inexperienced’ dyads.

Furthermore, since the relationship (in terms of rank distance and affiliative bond) between pack members in both wolves and dogs is known to affect tolerance in a feeding context^19,21^ and that both affiliation and dominance have been shown to affect success in a string-pulling tasks in a number of species^3,5,6,11,22^, we further assessed their role in cooperative success in the current study.

Overall, we predicted that, similarly to hyenas^11^, both wolves and dogs would benefit from being tested with an experienced partner, showing a higher success rate in comparison to non-experienced dyads. Nevertheless, in line with previous results using the same paradigm, we predicted that wolves would still outperform dogs, even when dogs were tested with an ‘expert partner’.

## Results

### Wolf-dog dyads comparison in success

**Figure 4:**
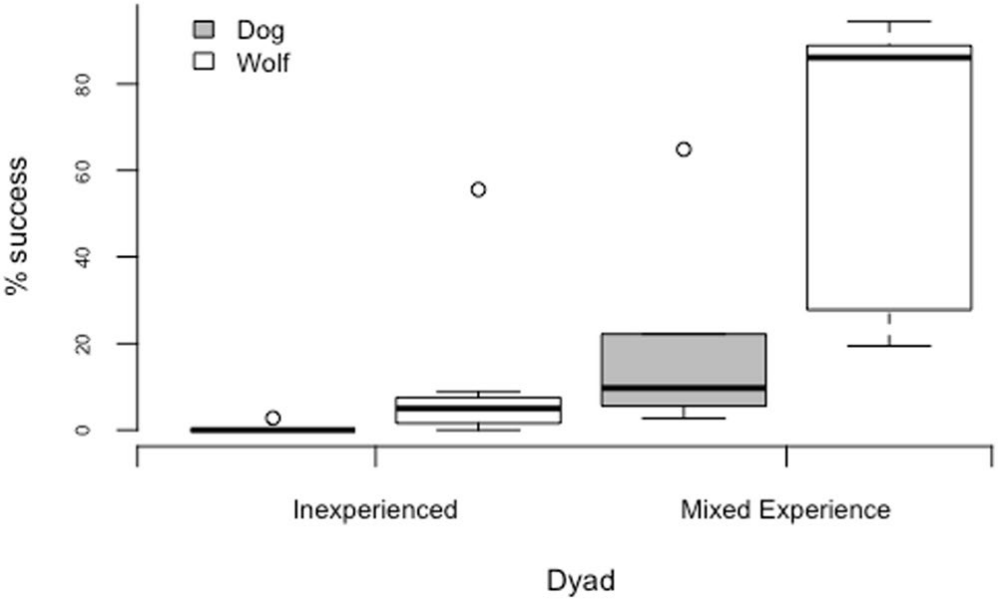
Mean success rate of inexperienced and mixed experienced wolf and dog dyads.

The mean success of inexperienced dog dyads (N = 8) was 0.3% (range 0–2.7% i.e. 0–1 trials), whereas mixed experienced dog dyads’ (N = 6) were successful on average in 19% of trials (range 3–65% i.e. 1–21 trials). The mean success of inexperienced wolf dyads (N = 7) was 11% (range 0–55.5% i.e. 1–25 trials), whereas mixed experienced wolf dyads’ (N = 5) succeeded on average in 63% of trials (range19–94%, i.e. 7–34 trials). Results showed that there was an effect of both dyad composition (mixed vs. inexperienced; GLM: χ^2^ = 17.21, p < 0.0001) and species (GLM: χ^2^ = 9.9, p = 0.002), but no interaction between these two variables (GLM: χ^2^ = 0.39, p = 0.533). In both wolves and dogs, mixed experienced dyads were more successful than inexperienced dyads, and regardless of dyad composition, wolves were more successful than dogs. In fact dogs’ performance with an experienced partner was more comparable to wolves’ performance in inexperienced dyads (see Fig. 4). Furthermore, of the 6 dog dyads tested with an experienced partner, only one dyad (Partner Nuru-Subject Pepeo) reached the criterion of succeeding in 4 out of 6 trials in two consecutive sessions, thereby progressing to the two-tray and delay condition (see^13^ for details of procedure). In the two tray-condition, in which two apparatuses were placed 10 meters apart in the same enclosure, the dyad succeeded in solving both trays on 77% (28/36) of trials across the 6 sessions presented. In the delay condition, in which the experienced partner was released 10 seconds after the subject, the subject (Pepeo) succeeded in waiting for the partner on 42% of trials.

### Effect of social relationships on success in dogs and wolves tested in mixed experienced dyads

Enough variation in success was observed when dogs were tested with an experienced partner to allow us to assess whether social relationships (affiliation and rank), as well as learning across sessions, affected the dyads’ performance. Despite the small sample size (N = 6 dyads) and therefore the limited reliability of results, we found that the higher the affiliation score (GLMM: χ^2^ = 15.81, p < 0.0001) and the higher the rank distance (GLMM: χ^2^ = 28.36, p < 0.0001), the more successful the dyad. Furthermore, success rates had a tendency to increase across test sessions (GLMM: χ^2^ = 3.4283, p = 0.064).

Interestingly, the success rate of mixed experienced wolf dyads (N = 5) was also positively affected by the dyads affiliation score (GLMM: χ^2^ = 18.09, p < 0.001), however the opposite rank effect was observed, with dyads with a smaller rank distance showing a higher success rate than those with larger rank distances (GLMM: χ^2^ = 4.31, p < 0.039). A strong effect of session (with an increase in success across session) was also observed (GLMM: χ^2^ = 11.35, p < 0.001).

Although neither for dogs nor for wolves, did we find a significant correlation between affiliation score and rank-distance (Spearman: dogs Rho = 0.13, p = 0.67, wolves Rho = 0.17, p = 0.59), it is interesting to note that in wolves the three individuals with the highest success rate had both the highest affiliation scores and lowest rank distances.

### Tolerance

In the first session in which they were tested, members of mixed-experienced dog dyads were more likely to simultaneously be active at the apparatus than members of inexperienced dyads (GLMM: χ^2^ = 7.94, p = 0.004). Furthermore, in mixed-experienced dyads, there was a trend for higher success in dyads that were more likely to be simultaneously active on the task (GLMM: χ^2^ = 3.58, p = 0.059). Interestingly, neither the likelihood nor duration of both animals being active on the task in mixed-experienced dyads differed between wolves and dogs (likelihood: GLMM: χ^2^ = 1.26, p = 0.26; duration: GLMM: χ^2^ = 1.22, p = 0.27). Looking at it in more detail, it emerges that whereas the expert dog spent more time active on the task than the expert wolf (GLMM: χ^2^ = 6.096, p = 0.013), there was no difference in the time spent active on the apparatus between the inexperienced wolf and dog in the dyad (GLMM: χ^2^ = 0.77, p = 0.38).

## Discussion

### The benefits of a task-experienced partner does not bridge the wolf-dog cooperative gap

In both wolves and dogs, being tested with a task-experienced partner positively affected the dyads’ performance. This suggests there may be a social learning effect and/or that the experienced individual, by adapting their behaviour to their less savvy partner, provided the dyad with the necessary opportunity to succeed, thereby allowing the inexperienced partner to learn from this success and show a better performance in subsequent trials. A similar result was found also in spotted hyenas tested in a version of the cooperative string-pulling task^11^.

Interestingly however, even if paired with an experienced partner, dogs were much less successful in coordinating their actions to obtain the reward compared to wolves. One could argue that the ‘expert’ wolves still had more experience than the ‘expert’ dogs, and in some respects this is indeed the case, since they had also negotiated the two-apparatus condition with a partner (which the dogs did not do). However, to become an ‘expert’, 2 of the 3 expert dogs were trained on a delay criterion much more stringent than the one set for the wolves, since they had to wait 30 seconds for their partner (rather than 10), and be successful on 8 out of 10 trials in two consecutive sessions (instead of just more than 40% success rate over all sessions). Hence by the time they had gone through training, we are confident that dogs had a comparable, if not greater experience of the task than the wolves. The other ‘expert’ dog, had no specific training, but successfully cooperated with its partner in the spontaneous, two-apparatus and delay condition, and therefore had the same experience as the wolves that were classified as experts.

Nevertheless *experienced* dog dyads hardly reached the cooperative success rate of wolf dyads in which both partners were *inexperienced*. Furthermore, only one dog dyad tested with the experienced partner reached the criterion allowing them to progress to the two-tray and then delay condition. In contrast, 12 out of 16 wolf dyads tested in the spontaneous and post-training conditions progressed to the two-tray and delay condition^13^. So it seems that being tested with an experienced partner still does not fill the gap between wolves’ and dogs’ cooperative abilities, which clearly emerged in both studies.

### How social relationships and tolerance affect cooperation

Although dogs’ success rates in the experienced partner condition were not overly impressive, the closer the affiliative bond and the higher the rank distance between dyad members, the better was their cooperative success in the string-pulling task. Given that the sample size was small, these results need to be taken with caution; however, the importance of affiliation in mediating cooperation in dogs is in line with studies of free-ranging dogs living in packs, in which individual participation in intergroup conflicts is more likely to occur the higher the number of affiliative partners the individual has in the pack^23^. Furthermore, in the same pack-living dogs tested here, the duration of peaceful food sharing in a dyadic experimental context was shown to be positively associated with the strength of the affiliative bond between partners^19^, suggesting that affiliation facilitates tolerance around a food source.

The influence of rank on cooperation is also in line with a number of previous studies in other species (macaques^5^; chimpanzees^3,4^; hyenas^11^), where it has been suggested that rank mediates cooperative success because it affects tolerance around a resource. In line with this, in free-ranging dogs, the greater the rank distance, the more tolerance at a communal feeding source was observed^21^.

Interestingly, although the relationship between affiliation and performance was the same in wolves as in dogs, the results on rank distance showed an opposite pattern with wolf dyads *closer* in rank showing a greater success rate than those with a larger rank distance. Again results need to be taken with caution given the small sample size, however the same pattern of results was observed in the larger dataset consisting of 12 dyads in the Two-tray condition^13^. Wolves’ and dogs’ socio-ecologies differ quite substantially, with wolves depending heavily on each other for hunting, whereas free-ranging dogs mostly rely on solitary scavenging on human resources (see^24^ for a review). Wolves’ pattern of participation in hunting parties tends to see the more senior pack members taking the lead in both the chase and the capture of the prey^25,26^. These animals are typically also the more dominant ones and those closest in rank. It is hence possible that the cooperative success in wolf dyads relates to the closer attention that animals with similar rank pay to each other. Whereas in dogs, success in such a task may be related more to tolerance; with animals more distant in rank being more tolerant of each other’s presence at the apparatus. However, this hypothesis will require further testing in the future.

One measure of the animals’ tolerance is the propensity for both animals to be close and active on the apparatus at the same time. Indeed, in our previous study, we found that wolf dyads were more likely to both be active on the apparatus compared to dog dyads. In the current study, when dogs were tested with an experienced individual, the likelihood of both being active on the task was significantly greater than in dyads composed of two inexperienced partners, and in the latter dyads the likelihood of both being active on the apparatus at the same time was no longer different from that of wolf dyads. Potentially such results are explained by ‘response facilitation’, whereby the activity of the experienced animal enhances the likelihood of the inexperienced one also being active on the apparatus, and possibly also increased tolerance by the dominant/experienced partner.

From food tolerance tests^18,19^ and from studies looking at post-conflict behaviour^20^ it emerges that dogs use avoidance, both when confronted with potential conflicts over food as well as after social conflicts, by maintaining a larger distance from each other. In contrast, wolves “argue” over food, and show affiliative behaviour (reconciliation) towards each other after a conflict. Similarly, in this cooperative task, dogs’ ‘avoidance strategy’, when close to a food source appears to limit their ability to work on the task together, but social factors, which reduce the likelihood of a conflict occurring, i.e. a good relationship (high affiliation score) and a larger rank distance (less competition), can facilitate proximity maintenance and simultaneous interaction on the apparatus and hence promote success.

Considering there was no wolf-dog difference in the likelihood of both being active on the apparatus at the same time when mixed experienced dyads were compared, tolerance alone does not however, seem to explain why wolves outperformed dogs in this condition. Wolves and dogs show similar performance in means-end understanding tests^27^, suggesting that at least the basic mechanical/physical aspect of the task should have been comprehensible to both species. However, one possibility that needs to be considered is that overall wolves acquired a better understanding of how the elements of the task fit together, and in particular of the need for a partner. In a recent study, wolves’ were shown to outperform dogs in their causal understanding of the relationship between objects^28^. In the current task, animals were required to understand the relationship between the presence of the partner and its actions with the success in moving the tray. It is hence possible that multiple factors are affecting dogs’ performance on the cooperative task, both their tolerance around a desired resource and their causal understanding between the elements of the task. Further testing will be needed to evaluate these aspects individually.

The current study aimed to investigate wolf-dog differences in intraspecific cooperation by using a classic experimental task used with many different species (see introduction). This type of comparison involving a novel apparatus and people present to prepare the experimental setup can only be considered unbiased when both species are equally at ease with such procedures, hence the use of hand-raised captive animals with comparable experience. Nevertheless, such a comparison inevitably carries other limitations since captivity may alter the reliance that pack members have on each other and/or the strength of their affiliative bonds. These factors may in turn affect their cooperative success. Future studies with free-ranging dogs living in groups of different sizes and in different environments would be highly desirable to further evaluate the social and ecological aspects, which may affect cooperative success.

### Conclusions

In sum, although being paired with an experienced partner significantly improved the dogs’ success in the string-pulling task, increasing their tolerance with each other, and hence allowing them greater chance at being successful, they nevertheless performed significantly worse than wolves. These results further support the view that a change in dogs’ socio-ecology, leading to a lifestyle based more on solitary scavenging and hence a reduced reliance on conspecific cooperative activities such as group hunting (and therefore also carcass-sharing) and pup-rearing, may have altered dogs’ communicative and conflict management styles with conspecifics and ultimately their cooperative inclinations. Future studies will be needed to establish whether changes in their socio-ecology have also affected dogs’ understanding of causal relationships, which may further hinder their performance in such tasks.

## Methods

### Subjects

#### General aspects

Wolves and dogs at the Wolf Science Center (www.wolfscience.at) were tested. The wolves originate from wild parks in America and Canada. The dogs are all mixed breeds and of the animals in the current study eight originated from shelters in Hungary and eight were bred at the Wolf Science Center from Layla/Nia and external, mixed breed males. All subjects were bottle-fed and later hand-fed by humans from the age of 10 days. The wolves and Hungary-shelter dogs were raised in peer-groups with continuous access to humans as social partners for the first 5 months of their life. After 5 months all individuals were introduced into packs of adult animals. The dogs born at the WSC received the same experience during the day (non-fraternal peer groups and human social interaction and feeding form the age of 10 days to 5 months), but were returned at night to their mothers’ packs and at 5 months some remained with their original home packs whilst others were introduced to non-family packs. As adults, packs live in large 2000–8000 m^2^ enclosures and voluntarily take part in training and various behavioral experiments on a daily basis (see^18^ for more details).

#### Wolves

Data from a total of 12 dyads, composed of 13 wolves (7 males, 5 females) belonging to 4 packs tested in the Spontaneous condition, were analysed categorizing the dyads based on the experience of its members (Tables 1). This dataset was a subset of the data presented in^13^. Dyads were either composed of two Inexperienced members i.e. neither individual had previously been successful in the delay condition with another partner, or they were categorized as a Mixed dyad i.e. composed of one inexperienced individual and one experienced individual who had been successful in the delay condition with another partner on more than 40% of trials (Table 2). This criterion was chosen because the crucial aspect of the cooperative string-pulling task is that the two animals have to pull simultaneously on the two rope ends. If one of them is delayed the other one needs to inhibit pulling the rope until the partner arrives. An animal that waits for a partner to arrive at the apparatus before pulling the rope is likely to have learned that coordinating actions with the partner is necessary for success, and can therefore be considered ‘experienced’. Nevertheless, we acknowledge that we cannot tease apart whether animals have acquired a set of associative rules (pull only when your partner is close) or whether success in the delay shows an understanding of the ‘cooperative nature’ of the task.

**Table 1:** Dyads tested in the Spontaneous condition, categorized in terms of the prior experience of dyad members; including number of trials completed and number of successful trials.

Dyad	Dyad experience	Affiliation score	Rank distance	N. of trials	N. successful trials
Amarok-Kenai	Inexperienced	1.08	0.43	32	1
Geronimo-Amarok	Inexperienced	3.45	4.29	37	0
Geronimo-Kenai	Inexperienced	0.50	4.71	36	0
Kaspar-Shima	Inexperienced	0.75	20.0	45	25
Nanuk-Una	Inexperienced	0.73	2	48	3
Tala-Chitto	Inexperienced	2.00	5.91	34	3
Wamblee-Yukon	Inexperienced	0.91	3.00	40	2
Chitto^*^-Shima	Mixed	1.23	5	36	32
Kaspar^*^-Tala	Mixed	1.37	9	36	34
Tala^*^-Shima	Mixed	0.41	10.9	36	10
Kaspar^*^-Aragorn	Mixed	1.09	5.9	36	31
Kaspar-Chitto^*^	Mixed	1.23	15	36	7

Four wolf dyads in which both partners were experts (but are not included in this dataset) showed success rates of 77%, 100%, 100%, 100%. *Hihglights the experienced partner in the dyad.

**Table 2:** Percentage of successful trials performed by the ‘experienced’ partner in the delay condition, prior to being tested with the inexperienced partner in the One-tray condition.

Individual	% success delay condition
Tala	61
Kaspar	64
Chitto	55.5

#### Dogs

A total of 14 dyads, composed of 16 dogs (9 males and 7 females), belonging to 6 packs were tested (see Table 3). Inexperienced dog dyads were part of the dataset published in^13^, whereas the Mixed experienced dyads were tested in the current study.

**Table 3:** Dog dyads, experience level, number of trials completed and number of successful trials.

Dyad	Dyad experience	Affiliation score	Rank distance	N. of trials	N. successful trials
Maisha-Binti	Inexperienced	2.483	na	32	0
Asali-Bora	Inexperienced	1.45	1.75	38	0
Meru-Nia	Inexperienced	0.43	0.88	33	0
Nuru-Zuri	Inexperienced	1.21	2.84	32	0
Layla-Zuri	Inexperienced	1.93	6.01	36	0
Nuru^$^-Layla^$^	Inexperienced	1.34	3.17	42	0
Imara-Hiari	Inexperienced	3	0.37	36	1
Sahibu-Gombo	Inexperienced	2.17	2.42	9+	0
Nuru^*^-Pepeo	Mixed	1.6	9.41	37	24
Pepeo^**^-Panya	Mixed	1.85	6.08	36	8
Nuru^*^-Panya^#^	Mixed	0.43	15.49	37	1
Pepeo^**^-Enzi	Mixed	1.61	1.61	18	1
Meru^*^-Hiari^##^	Mixed	0.01	4.13	36	5
Meru^*^-Imara^##^	Mixed	0.63	3.75	36	2

^$^Both Nuru and Layla had been tested in a previous dyad but had been unsuccessful + stopped in session 2 (after only 9 trials) because of serious risk of aggression. This combination could not be tested further.

^*^Experienced partner = most dominant member in the pack and trained with a pet dog until successful in the delay condition.

^**^Experienced partner = successful in the two tray and delay condition with Nuru (see method section).

^#^Previously tested with Nuru but successful only in 1 trial.

^##^Previously tested in the Spontaneous condition (Imara-Hiari dyad) but successful only in 1 trial.

In the case of dogs the Experienced partner was either (1) the most dominant pack member, trained to solve the task with a familiar non-pack member and perform successfully also when the partner was delayed (see below for details) or (2) a pack member who had been successful in the Spontaneous, Two-tray and Delay condition (see below for details) (Table 3).

#### Dyad relationship assessment

Daily observations of all pack members are carried out at the WSC by staff and students. Students are permitted to collect data independently only after inter-rater agreement observations with a senior member of staff show a Cohen Kappa greater than 0.75 (calculated on all behavioural categories, for each of 10 sets of observations in at least two packs of animals, using Noldus Observer version 10.5). Ten-minute focal animal sampling, focused on the social interaction with other pack members is carried out for each member of a pack, during different times of day. The Pocket Observer program (3.2 Software) is used for data collection then imported into the Observer XT 10.5 program (both from Noldus Information Technology, Wageningen, The Netherlands) for further analyses.

Based on such observations, we calculated (1) an ‘Affiliation score’ for each dyad, i.e. the bidirectional frequency of affiliative behaviour exchanged by individuals A and B, divided by observation time (hours) for subjects A + B; and (2) the rank distance between members of the dyad, i.e. subtracting the David’s score of individual A from that of individual B. The David’s score for each individual was calculated based on the frequency of dominant and submissive behaviours displayed towards other pack members. The David’s score is considered the most accurate measure of an individuals’ dominance status within the pack, since it takes into account the relative strength of all pack members, thereby also allowing an assessment of relative strength across groups with different numbers of members within^29^.

### Apparatus and test location

The apparatus consisted of a large (1.5 m × 75 cm) food delivery tray with a single rope passing through hoops in the tray, both ends lying on the ground. The tray was placed on the outside of the animals’ test enclosure, whereas the rope ends were positioned so as to go through the fence and lay on the ground inside the enclosure. The rope was 520 cm long, with 120 cm dangling from each end of the apparatus. Adjacent to each rope, 20 cm apart from each other, two food delivery areas were set, each containing one dead chick and one chunk of raw meat. To move the tray forward and obtain the food reward both individuals in the dyad needed to pull an end of the rope each at the same time.

**Figure 5:**
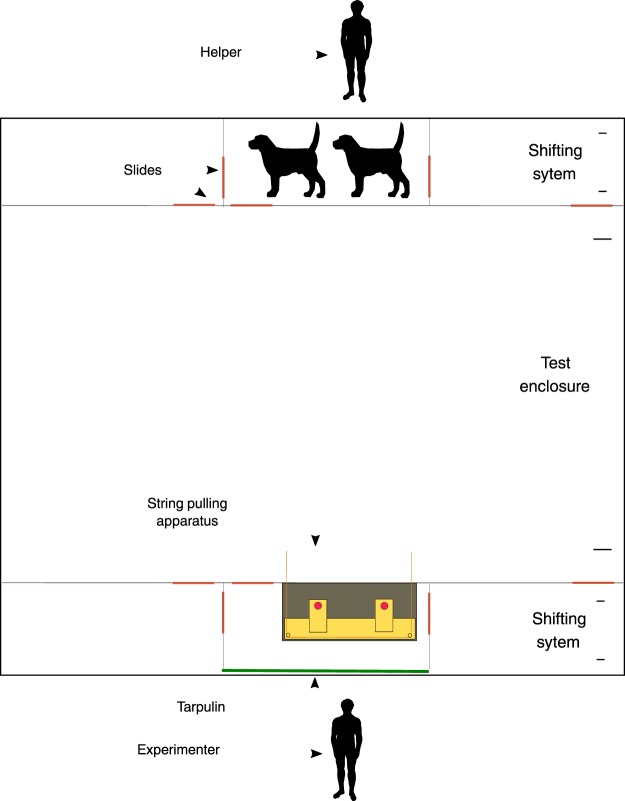
Experimental setup (dog and human image licensed under CC BY-SA 2.5 https://creativecommons.org/licenses/by-sa/2.5/deed.en, authored by: Abujoy; licence available at: https://commons.wikimedia.org/wiki/File:ComparaisonBeagle.svg. The image was not modified).

Testing took place in the two main testing enclosures at the Wolf Science Center. The starting location of the animals was on the opposite side of the testing enclosure 23 metres away from the apparatus (see Fig. 5). Before the test dogs were given 5 minutes to explore the empty enclosure. During testing the experimenter was behind a screen, out of sight of the animals. At the start of each trial the experimenter called the animals by name and showed them the food being placed on the apparatus.

### Overall procedure

Wolf and dog dyads were first tested in the Spontaneous condition, where they were given the possibility of interacting with the apparatus with no prior knowledge of it. If a dyad failed to solve the spontaneous condition, similarly to previous studies using the loose string paradigm^17^, individuals went through a training procedure whereby they learned that when ropes were placed close enough to each other, holding both in the mouth and pulling would allow them to solve the task (Individual string-pulling training). Following this training procedure, dyads were re-tested with a single apparatus (Post-training condition). Both wolves and dogs were tested in these two conditions^13^.

Wolf dyads that passed a criterion of 4 successful trials out of 6 in the last two sessions of either the Spontaneous or Post-training condition were then presented with a Two-tray condition, in which two apparatuses were presented in the same enclosure 10 meters apart. Finally, regardless of performance in this condition, wolf dyads were also presented with a delay condition, in which the subject was released into the test enclosure with the apparatus 10 seconds before the partner. The dog dyads did not meet the criterion necessary to move on to the Two-tray and Delay conditions (see Marshall-Pescini *et al*. 2017 for details).

As mentioned above, from the dataset published^13^, we selected ‘inexperienced’ and ‘mixed experience’ wolf dyads as well as ‘inexperienced’ dog dyads for evaluation in the current study. However, we needed to engineer ‘mixed experience’ dog dyads. To do so in the available dog packs the most dominant dog member was extensively trained to solve the string-pulling task together with a familiar non-pack member (pet dog), and to perform successfully with it in a 30 second delay condition. Pack-members were then tested on the string pulling apparatus (Spontaneous condition) either with the trained experienced partner (see Dog experienced partner) or a pack-member that had been successful in a two-tray and delay condition with the ‘experienced partner’. Procedures for Spontaneous, Two-tray and Delay conditions are fully described in^13^ and summarized here.

#### Spontaneous condition

A trial started with the experimenter calling the animals’ attention whilst holding food and visibly placing it on the apparatus. The two animals were then simultaneously released from their start location. Trials lasted 2 minutes, or ended either once the task was completed successfully and the animals had finished the food, or when the rope was pulled solely on one side, making the other end unavailable and hence making the tray impossible to move. At the end of each trial they were called back to the start position, whilst the experimenter set up the task again, for the following trial. If an individual never pulled the rope during the trial, motivation string pulling trials were presented (see details below) at the end of each session. All dyads were given an average of two sessions per week for a total of 6 to 8 sessions.

In session 1 and 2, animals were presented with 6 test trials. In sessions 3 to 6 the number of test trials was no longer fixed but varied from between 2 to 6 depending on the animals’ behaviour. If neither animal pulled the rope for two consecutive trials the session was stopped, to avoid frustration. All animals received motivation string pulling trials following these sessions (see details below). If in session 6 animals were presented with fewer than 4 trials, two additional sessions were presented. In sessions 7 and 8 dogs again received a fixed number of 6 trials, independent of their performance, followed by motivation string pulling trials if they did not pull the ropes for 2 consecutive trials. Overall, mean number of trials was 35 with a range between 32 and 42 (see Table 3), excluding one dyad that completed only 9 trials and had to be interrupted due to high intensity threatening behaviour.

*Motivation string pulling trials* varied depending on session. In all cases animals were allowed into the enclosure one at a time (i.e. without the partner). After sessions 1 and 2, a piece of meat was placed on the ground on the opposite side of the fence, out of the animal’s reach. The meat was attached to a rope, which the animal could pull to retrieve it. Following test trials in sessions 3 and 4 the food was placed on a wooden box outside the test enclosure with the rope dangling within the animal’s reach. These changes to the presentation were done to encourage the use of the mouth rather than the paw during string pulling. From sessions 5 to 8 in motivation string pulling trials, the meat was placed on the test apparatus, alternating the position of the food between the two food slots, with the meat directly attached to the rope. These changes to the presentation were carried out to further encourage animals to view the apparatus as a potentially reinforcing object, even if they had had no success during testing. The number of motivation string pulling trials depended on the animals’ performance in that as many trials were given as necessary for them to successfully retrieve the meat in three consecutive trials, with no prompting and/or encouragement.

#### Two-tray condition

If animals succeeded in at least 4 out of 6 trials in two consecutive session in the condition with one tray, the were presented with the two-tray condition. The two-tray condition involved animals being released into the enclosure at the same time, but with two apparatuses 10 meters apart form each other present in the test arena. Dyads were tested in 6 sessions, 6 trials per condition, and a trial was considered successful if animals obtained food from both apparatuses.

#### Delay condition

Regardless of performance in the Two-tray condition, subjects were presented with the delay conditions (6 sessions, 6 trials per session). In this case, the subject was released into the enclosure 10 seconds prior to the partner being released. The subject therefore had to wait for the partner to arrive at the apparatus before pulling on the rope.

The results for the two-tray and delay condition for wolf dyads is reported in Marshall-Pescini *et al*. (2017), dog dyads in that study did not pass criterion to continue in the other conditions. In the current study, one mixed experience dog dyad passed criterion and was presented with the two-tray and delay conditions (see results sections for description of their performance in these conditions).

### Dog experienced partner

#### Dog Experienced partner training

The most dominant individual in each of 2 packs was trained to solve the task with a pet dog owned by a Wolf Science Center trainer. The aim of this training was to allow the pack dog to gain sufficient successful experience of the apparatus with the pet dog, to potentially understand the underlying principle that another individual was necessary to solve the task.

To achieve this, the pet dog was first trained to pull the rope on the apparatus on command. In the *first training phase*, in each trial the two dogs were released into the enclosure at the same time. The trainer (positioned close to the apparatus) monitored the dogs’ behaviour and commanded her pet dog to pull the rope only when the pack dog was also pulling the other end of the rope, thereby facilitating the success of the dyad. Ten trials per session were carried out. The number of sessions depended on the pack dogs’ performance: when the pack dog was successful in 8 or more trials, he moved on to the second training phase.

The aim of the *second training phase* was for the pack-dog to learn to wait for its partner before pulling the rope. To achieve this aim, the two dogs were initially placed in separate starting locations and the pack-dog was always released before the pet dog. The trainer was located next to the apparatus and ten trials per session were carried out. The number of sessions depended on the pack dogs’ performance: we started with a 3 seconds delay and if the pack dog was successful in 8 or more trials, we increased the delay in the following session. Depending on the pack dogs’ performance we increased the delay in incremental steps of 2–3 seconds until pack dogs could wait for the partner in 8 or more trials out of 10 for 15 seconds. After the 15 second mark, we increased the time by 5–7 seconds increments until reaching a 30 seconds delay. When the pack-dog was successful in at least 8 of 10 trials at this delay, he moved on to the last training step.

In the *final training step*, the procedure was identical to the previous one (10 trials, 30-second delay) but this time, the experimenter and the trainer were behind the cover out of sight of the dogs. This step was carried out to confirm that animals would continue to perform the task without human social contact.

Training was considered complete when the pack dogs successfully waited for their partner on 8 or more trials in two consecutive sessions with a 30 second delay of the stooge partner. Once the experienced dogs were ready (Nuru and Meru), they were tested with their pack mates with a single apparatus, in 6 sessions consisting of 6 trials each.

In two dyads, the ‘Experienced partner’ (Pepeo) had not been trained as described above, rather, he had been tested with an experienced partner with the above training, and had then continued to be successful in a condition in which two-trays were presented, and in a delay condition where he was required to wait for 10 seconds for his partner (for a description of the two-tray and delay condition see^13^). In total he was successful in 100 of the 145 trials (69%) he was tested in across these conditions.

### Ethical statement

This study was discussed and approved by the institutional ethics and animal welfare committee at the University of Veterinary Medicine Vienna in accordance with Good Scientific Practice guidelines and all methods were performed in accordance with the relevant guidelines and regulations (Protocol number ETK-01/04/97/2014).

### Behavioural analyses

We coded the manipulation behaviour on the apparatus for both wolves and dogs in Session 1. We considered manipulation any interaction with the apparatus such as biting, pawing, mouthing the tray, rope or fence directly in front of the apparatus. Coding was done for each individual in the dyad but also taking into account what the other individual was doing, allowing us to establish the proportion of interactions with the apparatus in which both individuals were interacting with it simultaneously, vs. those in which one animal was interacting whilst the other was inactive.

### Statistical analyses

Wolf dyads were categorized in ‘Inexperienced’ and ‘Mixed’ based on the prior success rate of the individuals in the dyad when tested with other partners. These were a subset of the same dyads tested in^13^. Inexperienced dog dyads were also drawn from this dataset. Whereas Mixed experienced dog dyads were formed following the training of ‘experienced’ dogs.

To evaluate the role of species and prior experience in successful performance we ran a generalized linear model (GLM) with the number of successful trials (weighted by the total number of trials presented to the dyad) as the dependant variable, species (wolf vs. dog) and dyad type (Inexperienced vs. Mixed), and the interaction between these two variables as explanatory factors.

To evaluate the role of learning across sessions and the social relationship between the individuals in the dyad, we ran the analyses considering only Mixed experienced dyads (in which there was sufficient variability in the dogs’ performance to allow for this analyses to be conducted). We ran a Generalized Linear Mixed Model (GLMM) with the number of successful trials in each session (weighted by number of trials presented) as the dependant variables; session, affiliation score and rank distance as explanatory variables and dyad identity as random factor.

To explore the effect of tolerance on the likelihood of dog’s being successful in Session 1, we ran a (binomial) GLMM with the success in a trial as the dependant variable, and the frequency of both individuals in the dyad being simultaneously active on the apparatus in that trial as the explanatory variable, as well as dyad identity as the random factor. Furthermore, we ran a (binomial) GLMM, with the occurrence of simultaneous interaction by both dyad members on the apparatus as the dependant variable, dyad type (Inexperienced vs. Mixed) as explanatory factor and partner identity as random factor. The same model was also run with only Mixed experienced dyads and species as the explanatory factor.

A model reduction approach based on p-values was adopted. R statistics was used (version 3.2), packages ‘lme4’ for GLMM and ‘multcomp’ for corrected posthoc comparisons across conditions.

## Electronic supplementary material

Dataset 1

## Data Availability

All data generated or analysed during this study are included in this published article (and its Supplementary Information files).
